# Mental health measures during wildfires: the case of Ofunato

**DOI:** 10.3389/fpsyt.2025.1592688

**Published:** 2025-04-28

**Authors:** Yuka Kotozaki

**Affiliations:** Iwate Medical University, Morioka, Japan

**Keywords:** wildfire, mental health, PTSD, community resilience, disaster recovery

## Abstract

Climate change is increasing the threat of wildfires, causing severe physical, economic, and psychological impacts. This study analyzed the mental health effects of the February 2025 wildfire in Ofunato City, using government reports, media coverage, and disaster-related mental health studies. Stress, PTSD, social isolation, and economic hard-ship were identified as key issues, particularly among vulnerable groups such as the elderly and those with chronic illnesses. Economic instability from unemployment and delayed recovery may worsen these mental health outcomes. Effective interventions include immediate psychological support, long-term counseling, and rebuilding social connections. Collaboration among local governments, medical institutions, and support groups is essential to provide comprehensive mental health care, enhance community resilience, and support residents affected by wildfires’ recovery.

## 1 Introduction

Wildfires are among the most severe natural disasters (1, 2), affecting not only the environment but also society, the economy, and, crucially, human mental health (3, 4). The psychological impact of wildfires extends beyond the immediate trauma of evacuation and property loss; survivors often experience prolonged stress, anxiety, and post-traumatic stress disorder (PTSD) due to the uncertainty of rebuilding their lives (3, 4). With climate change contributing to rising global temperatures and increased drought conditions, the frequency and intensity of wildfires have escalated worldwide (2, 5). In Japan, although historically less affected by large-scale wildfires, has recently experienced an increasing wildfire risk (6). The February 2025 wildfire in Ofunato City exemplifies this growing threat. The large wildfires that occurred in Ofunato City, Iwate Prefecture, Japan, in February 2025, truly illustrate the serious impact of these wildfires (7, 8).

This article, written from the perspective of an external observer, examines the impact of the wildfires in Ofunato City on people’s mental health and proposes appropriate countermeasures based on available reports and data.

## 2 Wildfire in Ofunato, Iwate, Japan in February 2025

Ofunato City is located in the Tohoku region of Japan, on the southern coast of Iwate Prefecture, an area with rich forest resources and a beautiful natural environment of the rias coast (9). As of the latest census, Ofunato City has a population of approximately 70,000 residents, with a significant portion of the population residing in coastal and rural areas. The rias coast is characterized by its deeply indented shorelines, formed by the submergence of river valleys, creating a series of picturesque inlets and bays. This unique coastline is not only stunning in its beauty but also plays a vital role in the local ecosystem and economy (10).

Ofunato City was one of the hardest hit areas in Iwate Prefecture by the 2011 Great East Japan Earthquake. However, on February 19, 2025, a forest fire broke out in the Ryourihama area of Sanriku Town, Ofunato City, and spread rapidly due to strong winds; as of March 9, the fire was still not extinguished, and the area burned reached approximately 2,900 hectares, affecting approximately 9% of the total area of Ofunato (11).

The wildfire, the largest in Japan since 1990, caused widespread environmental damage and economic losses (12). Although human casualties were minimized, there are concerns about the respiratory effects of the smoke and the health risks to the residents (13). Residents faced the distressing reality of watching their homes burn, the fear of losing loved ones, and the uncertainty of whether they could return to a familiar life (14). Furthermore, the prolonged evacuation has caused psychological stress for many of the affected people (15). The Japanese government designated the large-scale wildfire that occurred in Ofunato City as a severe disaster, with the designation coming into effect on March 28, 2025 (13).

In this article, direct mental health assessments of residents affected by wildfires in Ofunato City are not included. Instead, the analysis is based on existing literature concerning the psychological impacts of similar disasters, particularly those related to wild-fires. While these findings provide valuable insights, it is important to acknowledge that the specific mental health effects in Ofunato may differ due to unique local factors.

## 3 The effects of wildfires on mental health

Wildfires can have profound and long-term effects on the mental health of affected. These impacts include stress, anxiety, PTSD, and social isolation, all of which can be ex-acerbated by economic hardship and prolonged recovery periods.

### 3.1 Psychological distress and PTSD

Immediately after a wildfire, residents living in areas where evacuation orders have been issued are forced to take emergency shelter (16). The inconvenience of living in shelters and the uncertainty of rebuilding homes and jobs are strong stressors for affected residents. Especially for those who have lost their homes, the lack of prospects for the future is a psychological burden (2, 4).

Exposure to life-threatening events, such as wildfires, significantly increases the risk of PTSD (2, 4, 17). According to previous study, 20-30% of disaster survivors may develop PTSD (4). In the case of the wildfires in Ofunato, many affected residents witnessed the flames spreading rapidly through the mountains or were forced to take emergency evacuation (18), and may be prone to flashbacks, insomnia, and emotional instability.

### 3.2 Social isolation and loneliness

Prolonged evacuation disrupts community ties and increases the risk of social isolation, especially for vulnerable groups such as the elderly and disabled (19–21). In post-disaster environments, those living alone or in temporary housing often struggle with loneliness and depression, exacerbating their psychological distress (22–24).

### 3.3 The effects on the mental health of affected residents due to post-fire economic hardship and delays in recovery

Post-fire economic hardships and delays in reconstruction also have a long-term effect on the mental health of the residents (25, 26). Anxiety disorders and depression have been observed to increase in past disasters as a result of prolonged job loss and housing reconstruction (25, 27–29).

## 4 Mental health care

Given the significant mental health impact of wildfires, it is essential to implement effective interventions. The following strategies can support affected residents.

### 4.1 Early intervention and psychological first aid

Immediately after a wildfire, Psychological First Aid (PFA) is a critical intervention to stabilize affected individuals and prevent long-term psychological trauma (30). PFA is a set of evidence-based techniques designed to provide immediate emotional and practical support to disaster survivors. It focuses on reducing initial distress, ensuring safety, and helping individuals connect with ongoing support resources (30). Ofunato City is currently providing the following support in cooperation with medical institutions (31).

1. Ensure safety: Provide a safe evacuation environment for affected residents.
2. Assistance with basic needs: prompt provision of food, water, and medical assistance.
3. Provide accurate information: publicize the fire situation and recovery efforts.
4. Facilitate social support: help maintain family and community connections.

### 4.2 Counseling and psychological support

Long-term mental health care is essential for wildfire survivors. Effective strategies include:

1. Professional psychological counseling:

Providing access to trained therapists and mental health professionals (4, 25).

1. Group therapy sessions:

Creating safe spaces for survivors to share experiences and support each other (32, 33).

1. Crisis hotlines and online consultations:

Establishing remote mental health support services for those unable to access in-person counseling (29, 30).

### 4.3 Community reconstruction and social support

Rebuilding of local communities will be essential as affected residents are forced to live in evacuation shelters for extended periods. Encouraging social reintegration through gatherings and support networks can help alleviate psychological distress, reduce isolation, and promote recovery, fostering a sense of normalcy (34, 35). Various international case studies provide valuable insights into community recovery after wildfires. However, it is crucial to adapt these examples to the unique circumstances of Ofunato City, taking into account differences in cultural, social, and governmental structures. These international examples can guide the development of effective strategies for Ofunato’s recovery, but careful consideration of local contexts will be key to their successful implementation.

#### 4.3.1 Organizing community events

In the aftermath of wildfires, community gatherings and support networks help restore social bonds and reduce psychological distress (34, 35). In a case study from California, USA, a local non-profit organization established Hope Plaza after the 2018 Camp Fire to prevent affected residents from becoming isolated. This temporary community space allowed affected residents to gather, share experiences, and support one another, significantly reducing psychological stress (36, 37).

Although Ofunato City’s population density and community structures differ from those of California, a similar approach could be beneficial. Establishing a community exchange space within temporary housing areas, along with regular resident meetings and counseling sessions, could facilitate social reintegration, helping residents maintain emotional well-being during their post-disaster recovery.

#### 4.3.2 Mobilizing volunteers

Volunteer-based mental health support has proven effective in post-disaster recovery worldwide (38, 39). After the 2009 Black Saturday fires in Victoria, Australia, the state government and a non-profit organization partnered to launch Bushfire Recovery Victoria, a disaster volunteer program. Volunteers with expertise in mental health and reconstruction provided direct assistance, helping affected residents re-cover both emotionally and physically (39, 40).

Ofunato City benefits from strong collaborative ties between local government, universities, and community organizations, which could facilitate the mobilization of mental health support volunteers. By bringing together psychiatrists, clinical psychologists, and trained community support volunteers, a structured mental health care system could be developed to address the unique needs of disaster survivors in Japan.

#### 4.3.3 Employment and financial assistance

Economic stability is a critical component of psychological recovery after disasters (41, 42). Following the 2017 wildfires in British Columbia, Canada, the provincial government created the Wildfire Recovery Jobs Program, allowing affected residents to participate in reconstruction work and infrastructure development. This initiative helped them regain financial independence while alleviating stress and uncertainty (43, 44).

Although Japan’s employment and social support systems differ from those in Canada, a similar post-disaster employment program could be developed in Ofunato City. By integrating affected residents into fire recovery infrastructure and forest conservation projects, affected individuals could rebuild their livelihoods while also benefiting from a structured recovery process that provides psychological and economic stability.

Rebuilding communities after wildfires involves more than just restoring infrastructure; it requires a comprehensive approach that includes social reintegration, mental health support, and economic recovery. The case studies from California, Australia, and Canada offer valuable insights into effective recovery strategies. However, it is crucial to recognize that each community, including Ofunato, has its own unique characteristics. Adapting these strategies to fit Ofunato’s specific cultural, social, and governmental context is vital for ensuring a tailored and effective response. By carefully considering the local needs and resources, Ofunato can implement the most suitable practices from these international examples, ensuring a more efficient and sustainable recovery process.

### 4.4 Mental health education and disaster preparedness

Preventive mental health education is critical in wildfire-prone areas (30, 45). Public awareness campaigns should integrate psychological resilience training into disaster preparedness programs (46, 47). Schools, workplaces, and community centers can play an active role in promoting mental health literacy and stress management techniques (48, 49). Currently, in Ofunato City, local authorities are providing basic shelter and immediate support to residents. However, further steps are recommended, such as the establishment of dedicated mental health support programs, which could complement the ongoing efforts.

## 5 Limitation

A limitation of this study is the reliance on extrapolated data from previous disaster research rather than direct assessments of Ofunato affected residents. While general pat-terns of wildfire-related psychological effects have been established in past studies, individual and community-level responses may vary. Future research should incorporate direct surveys or interviews with affected residents to obtain more localized in-sights.

## 6 Conclusions

The February 2025 wildfire in Ofunato City, still ongoing, may have had a significant impact on the mental health of evacuees. Psychological support in the immediate after-math of a disaster, long-term counseling, and the rebuilding of social ties can help alleviate the emotional burden on affected residents. Although the recommendations provided are drawn from global case studies, they are specifically tailored to address the unique needs of Ofunato City, which was significantly impacted by the 2011 Great East Japan Earthquake and subsequent disasters. By incorporating these recommendations into the recovery process, Ofunato City can enhance its community resilience and mental health support structures. By working together, local governments, medical institutions, and support groups can implement comprehensive mental health care that will help affected residents recover and improve the resilience of the entire community. It is crucial that these efforts are not delayed; immediate and coordinated action is essential to mitigate the long-term psychological impact and ensure a stronger, more resilient future for the affect-ed affected residents. Future research should incorporate direct assessments to develop more targeted mental health interventions.
